# Synergetic adsorption–photocatalysis process for water treatment using TiO_2_ supported on waste stainless steel slag

**DOI:** 10.1007/s11356-022-18728-8

**Published:** 2022-02-02

**Authors:** Eva Jimenez-Relinque, Siaw Foon Lee, Lorenzo Plaza, Marta Castellote

**Affiliations:** Eduardo Torroja Institute for Construction Science, IETcc, CSIC, Serrano Galvache 4, 28033 Madrid, Spain

**Keywords:** Metallurgical, Stainless steel slag, Titanium dioxide, Adsorption, Photocatalysis, Dye wastewater

## Abstract

This study presents an economical and efficient method to decolourise dye wastewater using industrial waste stainless steel slag (SSS). Titanium dioxide was immobilised on SSS by a precipitation–calcination method. Samples with different TiO_2_ loadings (prepared using either titanium isopropoxide precursor or commercial TiO_2_ nanoparticles) were used to decolourise an organic contaminant (methylene blue) under dark and UV conditions in aqueous solution, and their adsorption and photocatalytic performances were compared. Samples with 15 and 25 TiO_2_ wt% prepared by the precursor method had normalised photocatalytic efficiencies per gram close to that of bare TiO_2_; using an adsorption–photocatalysis process led to efficiencies 4.4 and 1.6 times higher than that of pure TiO_2_. The improvement in catalytic performance (greater for samples with less than 50% TiO_2_ content) may be due to better UV absorption ability (related to with the improvement of TiO_2_ particle dispersion) and the close TiO_2_ support interaction, which can eventually cause a photocatalysis-enhancing shift towards more negative oxidation potentials. The SSS also acted as an efficient adsorption trap for organic compounds. The pollutant was thus transferred to the TiO_2_ surface and photodegraded more rapidly and efficiently. The outstanding synergetic adsorption–photocatalysis capacities of TiO_2_ waste stainless steel slag composites for dye water treatment made the proposed conversion approach have great potential in practical applications.

**Graphical abstract:**

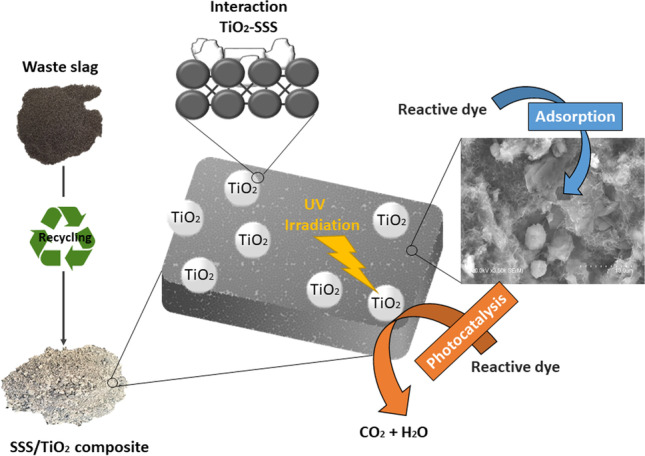

**Supplementary Information:**

The online version contains supplementary material available at 10.1007/s11356-022-18728-8.

## Introduction

Stainless steel production is one of the most dynamic sectors in the manufacturing industry. The world production of stainless steel in 2019 exceeded 52.2 Mt (data reported by the International Stainless Steel Forum, ISSF). Stainless steel is a recyclable material; nevertheless, for every 2 to 4 t produced, approximately 1 t of slag is generated (Das et al. 2007). The large amount of waste generated is not only an inconvenience, but also an environmental risk. New technologies for waste slag reuse must be developed to achieve a sustainable metallurgical industry.

Water pollutants, mainly from the textile industry, also affect environmental conditions adversely: they generate huge volumes of dye mixture wastewater. There are more than 10,000 types of commercially available dyes, and 5–15% end up in the waste stream (Li et al. 2020; Plaza et al. 2021). The increasingly restrictive enforcement of environmental regulations concerning the control of pollution from industrial effluent waste streams has made reliable remediation of heavily polluted waters a major research objective.

In particular, TiO_2_ photocatalytic processes, in which highly reactive photogenerated oxidative radicals completely eliminate a broad range of pollutants in aqueous media, have generated intense interest (Benabbou et al. 2007; Jiménez-Relinque et al. 2019; Jimenez-Relinque et al. 2016; Reddy et al. 2016, Yang and Wang 2018). However, there are barriers to the large-scale application of such methods, notably the agglomeration and the necessity of recovering the fine particles of TiO_2_ powder from the slurry stream (Foo and Hameed 2010). This problem could be avoided by immobilising the photocatalyst on a suitable supporting matrix to facilitate dispersion and subsequent recovery.

The combination of adsorbent substrates and photocatalysts such as TiO_2_ nanoparticles can enhance the catalytic performance. Adsorbent centres bring contaminant molecules close to the catalytic sites, thus elevating the degradation rate and capacity. In addition, there is a growing trend in interest and demand for nano-adsorption technologies in wastewater treatment processes. Because of their high surface area-to-volume ratio and surface multifunctionality, nanomaterials have grown in popularity in water treatment. Silica nanoparticles, a popular adsorbent, are used to remove various types of pollutants from industrial wastewater (Li et al. 2020). Silica can also be easily synthesised from the agricultural by-product rice husk (Rafiee et al. 2012). The use of low-cost adsorbents containing industrial or other by-products has advantages in terms of water treatment economy and environmental protection (De Gisi et al. 2016; Xue et al. 2022).

The active adsorption effect of natural clay combined with photocatalysis (Mishra et al. 2018) and fly ash–TiO_2_ mix (Shi et al. 2011) has already been studied. In the present study, we will consider the application of steel slag. Owing to its porous structure and high surface area, mechanical strength, and basicity, steel slag has a strong adsorption capacity, ion exchange ability and other characteristics useful in wastewater treatment (Plaza et al. 2021; Repo et al. 2015). Other previous work outlined the fabrication of TiO_2_-supported high surface area calcium silicate hydrate from blast furnace slags (Shi et al. 2017). However, this required prior acid–base activation treatment, decreasing production yield and increasing cost. No study has reported the fabrication of composite stainless steel slag–TiO_2_ (SSS/TiO_2_) or its application in adsorption and photocatalytic degradation for wastewater treatment.

The present study reports a novel and efficient recycling route for preparing stainless steel slag–TiO_2_ (SSS/TiO_2_) composites with different TiO_2_ contents, as well as their efficiency as adsorbent photocatalyst hybrids of methylene blue (MB) in aqueous effluents. The hydroxyl radical formation rate in photocatalytic process was also measured as an indicator for evaluating the photocatalytic performance by photoluminescence using terephthalic acid as a probe molecule. The crystalline composition, morphology, electronic band structure and light absorption capacity of the composites were also evaluated.

## Materials and methods

### Materials

The SSS material was provided by Acerinox Europa, SAU, Spain. The chemical composition of SSS used was CaO, 44.38; SiO_2_, 28.41; MgO, 13.3; Al_2_O_3_, 5.59; Fe_2_O_3_, 2.56; Cr_2_O_3_, 2.44; TiO_2_, 1.4; and MnO, 1.37 (wt%), with other minority components.

#### Raw SSS leaching

Leaching experiments were performed on raw SSS according to the EN 12,457–2 standard. These consisted of batch leaching powdered slags using deionised water (10 L/kg) under continuous stirring at room temperature for 24 h. The amount of leached metal was determined using inductively coupled plasma spectroscopy.

#### *SSS/TiO*_*2*_* composite preparation*

SSS/TiO_2_ composites with different TiO_2_ mass contents were prepared by two precipitation–calcination routes and denoted *X*M*Y*, where *X* represents the TiO_2_ mass percentage (15, 25, 50, 75, 100) and M*Y* (*Y* = 1,2) represents the method used.

In method 1 (M1), the necessary amount of SSS was dispersed in 50 ml of ethanol, followed by the dropwise addition of 5 ml of titanium isopropoxide (TTIP) (Bengtsson et al. 2009). The mixture was then shaken and heated at 65 ± 5 ºC until the solvent had completely evaporated. SSS/TiO_2_ was calcined at 500 °C for 24 h at 20 °C/h.

In method 2 (M2), the SSS was mixed with commercial TiO_2_ nanoparticles (Evonik Aeroxide® P25) in 20 mL of isopropanol. The mixture was sonicated for 30 min, followed by stirring for 2 h. The solvent was removed at 65 ± 5 ºC. Finally, the products were calcined at 400 °C for 6 h at 20 °C/h.

### Composite characterisation

The chemical composition of the samples was analysed by X-ray diffraction (XRD) using a Bruker D8 Advance diffractometer, and the microstructure and morphology were analysed by scanning electron microscopy with energy-dispersive X-ray spectroscopy (SEM–EDX) (JSM-6700F system). Adsorption isotherms for N_2_ were obtained using an ASAP2010 analyser to measure the specific surface area and pore size distribution of the materials. Diffuse reflectance spectra (DRS) were recorded using a UV-2600 Shimadzu spectrophotometer to characterise the UV–visible absorption spectra and band edges (Jimenez-Relinque et al. 2017). Mott–Schottky analysis to obtain the band flat potentials was conducted at 400 Hz with a sinusoidal signal amplitude of ± 10 mV. SSS–TiO_2_ powder and hydroxyethyl cellulose paste were coated on fluorine-doped SnO_2_ (FTO) glass (6–9 Ω/square). The electrochemical cell consisted of FTO-SSS/TiO_2_, Pt wire, Ag/AgCl and 3 M KCl as the working electrode, counter electrode and reference electrode, respectively, in 0.2 M Na_2_SO_4_ at pH 6.5.

### Adsorption and photocatalysis tests

Two types of process were investigated: the AD process (MB dye adsorption on the SSS/TiO_2_ substrate in dark conditions) and the PH process (MB photocatalytic degradation under UVA light irradiation (7.5 W/m^2^, λ_max_ = 360 nm)). In a typical test, sealed glass vessels containing 100 mg of SSS/TiO_2_ composites dispersed in 10 mL of MB solution (15 ppm) were placed on a rotator at 50 rpm. At different times, aliquots of the suspension were collected and centrifuged at 4000 rpm for 15 min. The dye concentration in the supernatant was determined by measuring the absorbance at 668 nm using a Shimadzu UV–Vis spectrophotometer. The discolouration percentage of MB at each time point was calculated. Raw SSS and bare TiO_2_ were also evaluated for comparison.

The photocatalytic activity of the as-prepared samples was also assessed using the terephthalic acid (TA) fluorescence (FL) probe method (Ishibashi et al. 2000, Jimenez-Relinque and Castellote 2015). TA reacts with OH• photogenerated by photocatalysis to produce a highly fluorescent product, 2-hydroxyterephthalic acid (TAOH; λ_exc_ = 315 nm, λ_em_ = 425 nm). The fluorescence change during the irradiation time provides proof of the photocatalytic activity of the material (Hirakawa and Nosaka 2002, Jimenez-Relinque and Castellote 2015). In this study, 0.2 g of SSS/TiO_2_ were suspended in 20 mL of TA (2 mM) aqueous solution containing NaOH (2 mM) in sealed glass vessels. The fluorescence intensity of the solutions was measured at different irradiation times using a fluorescence spectrophotometer (Perkin-Elmer, LS-55).

## Results and discussion

### Leaching test

Table S1 shows the pH, conductivity and concentration of different elements in the collected leachate. For comparison, the table also includes limits from the Italian Ministerial Decree 2006/2018 (which specifically regulates the recovery and reuse of steel slag as a recycled aggregate for concrete production (Sorlini et al. 2012)) and two other European regulations (Dinçer et al. 2007). All results were much lower than the regulatory criteria require.

### Composite characterisation

**Fig. 1:**
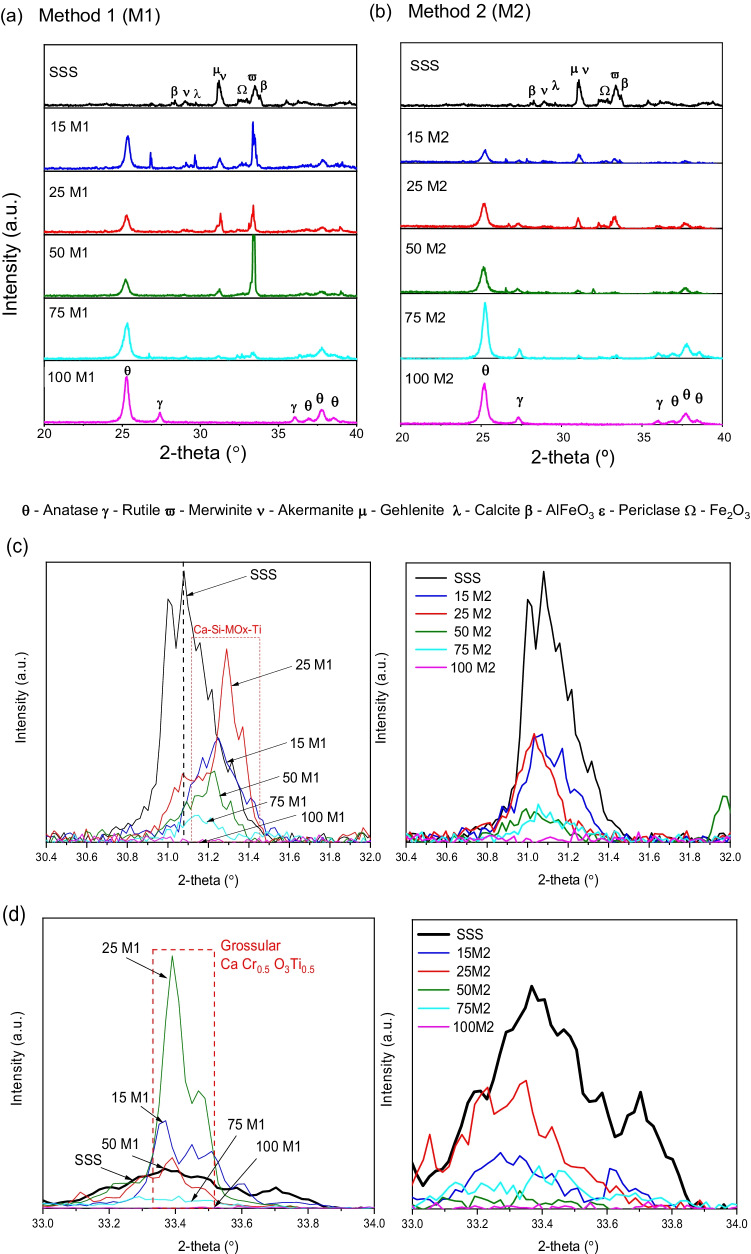
**a**–**b** X-ray diffraction spectra of raw stainless steel slag (SSS) and synthesised SSS/TiO_2_ composite samples by methods **a** M1 and **b** M2. **c** Formation of Ca–Si-MOx-Ti (MO = metal oxides) in samples prepared by each method. **d** Evidence of CaCr_0.5_O_3_Ti_0.5_ and grossular (AlCa_2.8_ Fe_0.95_ Mn_0.04_O_12_ Si_3_Ti_0.12_) in samples prepared by M1 but not by M2. Samples are identified by percentage of TiO_2_ followed by method name (e.g. 50M1).

The XRD patterns of the analysed samples are shown in Fig. 1a–b. Raw SSS presents diffraction peaks corresponding to mineral phases such as merwinite (Ca_3_Si_2_MgO_8_), akermanite (Ca_2_Si_2_MgO_7_), gehlenite (Ca_2_Al(SiAl)O_7_), cristobalite (SiO_2_), calcite (CaCO_3_) and periclase (MgO), as well as some metallic mixtures such as AlFeO_3_ and Fe_2_O_3_. Cr-containing phases, mainly in the form of spinel, were detected. In pure TiO_2_ synthesised by M1 (100M1), anatase and rutile crystalline phases were formed. The XRD pattern of the 100M2 sample confirmed the presence of anatase along with the rutile phase, as certified by the catalyst’s producer company. The XRD pattern of the SSS/TiO_2_ composite samples prepared by M1 contained anatase but not rutile, indicating a differential conformation of TiO_2_ in the presence of SSS. By contrast, the composites synthesised by M2 maintained both crystalline TiO_2_ polymorphs. In addition, the XRD pattern of the composites synthesised by M1 showed junctions between the Ti and SSS phases. As an example, in Fig. 1c, it can be seen that from the akermanite phase initially present in the SSS, the inclusion of Ti in the crystal network results in modified phases Ca–Si-MOx-Ti (where MO means metal oxides), in agreement with Shi et al. (2017). In addition, new, mostly titanium-containing phases such as Ca-Cr–O-Ti, grossular, RhTi, TiSi_2_ and Al-Ni-Zr are formed, particularly in the samples with TiO_2_ loadings between 15 and 50%. An example is contained in Fig. 1d, where the formation of a phase containing Ca, Cr, Ti and grossular (more intense for 50M1) can be seen. In the case of samples synthesised by M2 (Fig. 1c, d), there is no indication of a combination of TiO_2_ and SSS compounds.

**Fig. 2:**
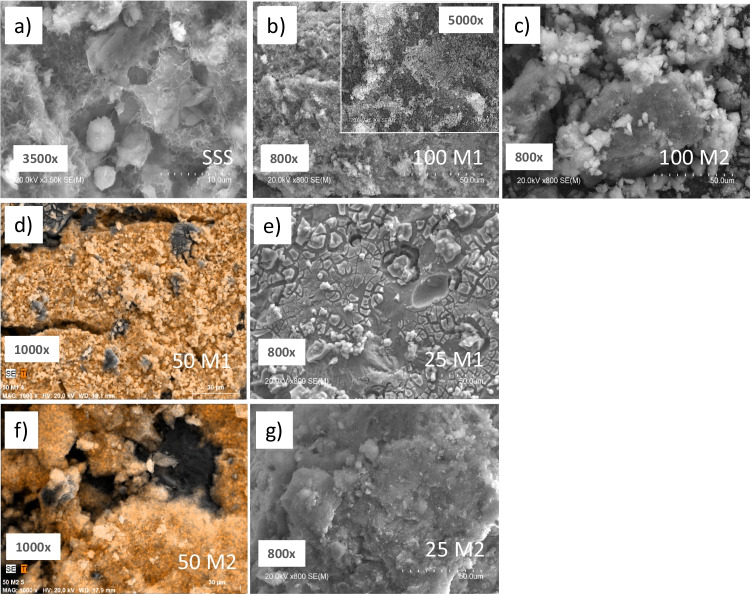
SEM–EDX images of **a** stainless steel slag, **b** 100M1 nanoparticles, **c** 100M2 nanoparticles and various SSS/TiO_2_ composite samples from **d**–**e** M1 to **f**–**g** M2. (M1, M2 = synthesis method.) Samples are identified by percentage of TiO_2_ followed by method name (e.g. 50M1)

Figure 2 shows the SEM–EDX characterisation of the samples. SSS displays an open framework structure that might be useful for Ti deposition and the adsorption of organic pollutants. 100M1 consists of roundish agglomerated nanoparticles only distinguishable at 5000 × magnification. The 100M2 nanoparticles are roundish agglomerates with sizes of 30–50 nm, as certified by the producer. Some significantly larger structures also appear, probably resulting from nanoparticle agglomeration during synthesis. Both methods result in an SSS surface well-covered by TiO_2_, but the morphology depends on the preparation method. M1 led to TiO_2_ flack structures and some roundish agglomerates deposited onto the SSS surface, indicating the close combination of TiO_2_ and SSS support. M2 led to a massive structure; it was not possible to distinguish between the TiO_2_ sintered nanoparticles and the SSS support surface. These results agree with the XRD results describing the interaction of TiO_2_ and SSS compounds in terms of the synthesis method.

The microstructural parameters obtained by BET analysis are listed in Table 1. The surface area of SSS/TiO_2_ composites synthesised by M1 did not show a linear dependence on the TiO_2_ loading; the specific areas of samples 25M1 and 50M1 were clearly higher than that of SSS. A clear decrease in the mean pore diameter (especially at < 50% TiO_2_) was observed. This may be due to the precursor of TiO_2_ filling the internal pores of SSS during the synthesis process. The composites synthesised by M2 showed a higher surface area and pore volume as the TiO_2_ load increased. This may indicate that, during M2, the commercial nanoparticles were not combined and did not fill the pore structure of the SSS support.

**Table 1:** TiO_2_ mass contents and structural properties of samples prepared by method 1 and method 2

Sample	TiO_2_ content (%)	BET (m^2^/g)^a^	V_p_ (cm^3^/g)^b^	D (nm)^c^
SSS	0	3.47 ± 0.02	0.018	20.3
*Method 1 (M1)*				
15M1	15	3.03 ± 0.03	0.009	11.9
25M1	25	7.12 ± 0.05	0.020	11.4
50M1	50	10.45 ± 0.08	0.026	10.0
75M1	75	2.78 ± 0.01	0.004	15.2
100M1	100	12.36 ± 0.15	0.045	14.4
*Method 2 (M2)*				
15M2	15	6.53 ± 0.05	0.044	27.0
25M2	25	11.96 ± 0.10	0.066	21.9
50M2	50	23.59 ± 0.16	0.097	16.4
75M2	75	33.45 ± 0.29	0.166	19.9
100M2	100	47.03 ± 0.59	0.285	24.2

^a^Surface area determined by BET method; ^b^pore volume defined at P/P_0_ = 0.99; ^c^pore diameter determined from the BJH pore size distribution curve.

**Fig. 3:**
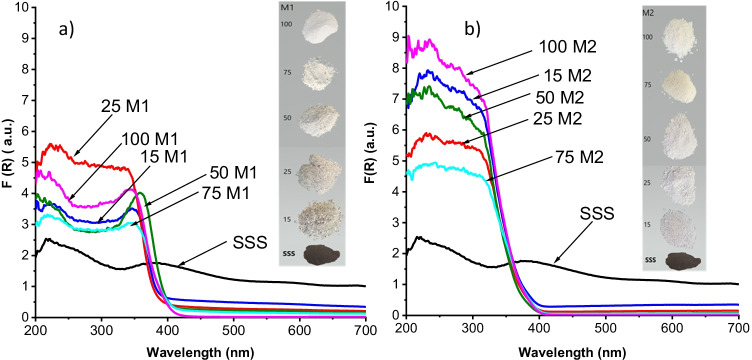
UV–visible diffuse absorption spectra of samples prepared by methods **a** M1 and **b** M2, with images of the samples. Samples are identified by percentage of TiO_2_ followed by method name (e.g. 50M1)

**Fig. 4:**
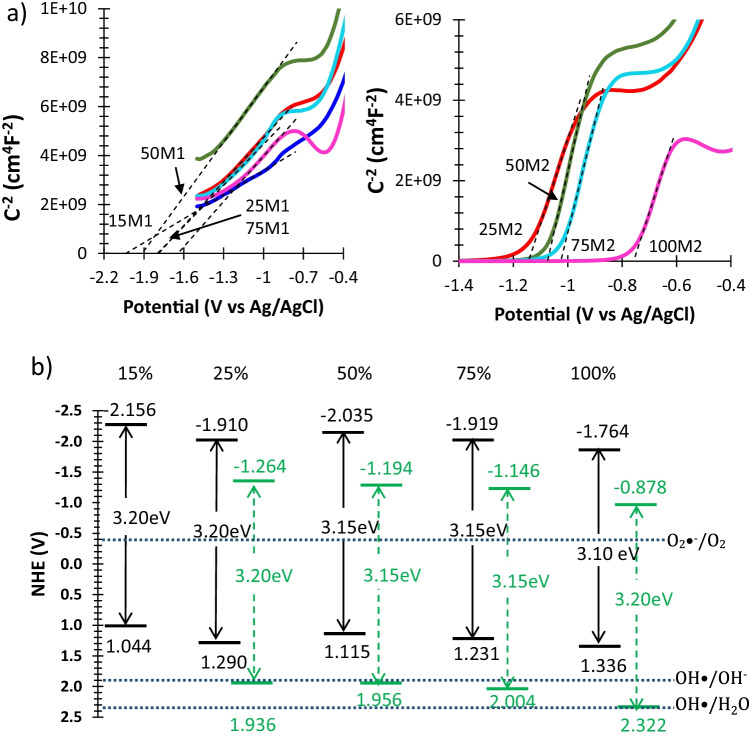
**a** Mott–Schottky plots for synthesis methods (left) M1 and (right) M2. Samples are identified by percentage of TiO_2_ followed by synthesis method name (e.g. 50M1). **b** Schematic diagram of the band structure of M1 (black) and M2 (green dashed) samples with different TiO_2_ loading.

The UV–visible absorbances of the samples are shown in Fig. 3. The raw SSS shows lower UV and higher visible absorbance (due to its black appearance) than the composite samples. All SSS/TiO_2_ samples show a strong absorption between 200 and 350 nm, consistent with the characteristic band–band transition of TiO_2_ but do not exhibit a noticeable absorbance in the visible range; this is attributable to the good coverage of the SSS support, even in those samples with lower TiO_2_ content. The absorption for 25M1 is even higher than that of 100M1, suggesting better dispersion of the TiO_2_ on the SSS support. In the case of M2, the highest absorption corresponds to that of bare TiO_2_. The band edge (E_g_) values obtained from these results are presented graphically in Fig. 4b.

Figure 4a shows the Mott–Schottky plots for the M1 and M2 samples. The intercept on the *x*-axis gives the flat-band potential of each sample. The procedure for calculating the conduction band (CB) and valence band (VB) from the flat-band potential approach is given in detail in Giannakopoulou et al. (2017) and van de Krol et al. (1997). The CB and VB structures of the M1 and M2 composite samples on the normal hydrogen electrode scale are shown schematically in Fig. 4b. The standard energy potentials for O_2_/O_2_•^−^ (–0.33 V), H_2_O/OH• (2.33 V) and OH − /OH• (1.9 V) are also included.

Samples of M1 show an upward shift in band energies compared to samples of M2. The introduction of SSS does not produce any clear trend in the E_g_ values except a shift towards negative potentials compared to bare TiO_2_. This is in accordance with the lower photocatalytic oxidation capability of SSS/TiO_2_, as will be discussed later. For M2, the shift is larger as the amount of SSS increases. M1 also showed this behaviour, with the peculiarity that sample 25M1 has the highest oxidation capability of the M1 composites. The VB levels of the M1 samples are not positive enough to induce the generation of OH• from adsorbed H_2_O or OH-, but their CB levels are negative enough for O_2_•^−^ production. For M2 samples, the VB level is closer to the oxidation potential of H_2_O and OH-, enabling the direct formation of OH• by oxidation. The CB levels of M2 samples are negative for O_2_•^−^ formation.

### Adsorption and photocatalytic tests

**Fig. 5:**
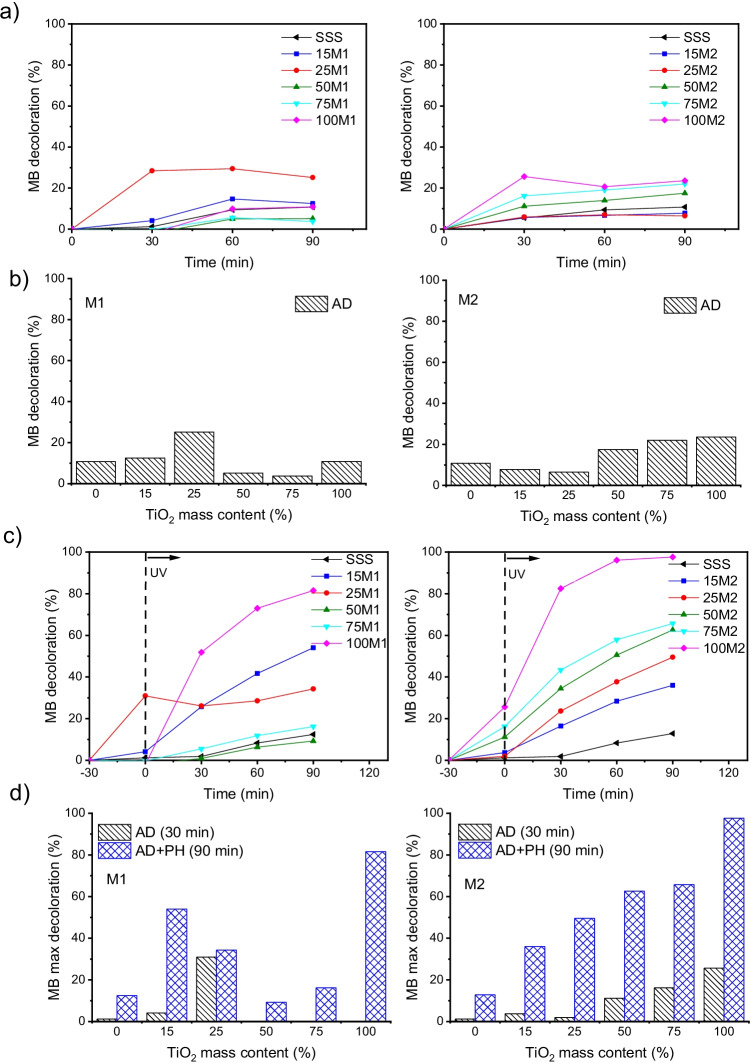
**a** Degradation profile of methylene blue (MB) in dark in the presence of samples with different TiO_2_ mass contents. **b** The dependence of MB decolouration (%) on TiO_2_ mass content (%) by adsorption (AD). **c** Degradation profile of MB in dark conditions (30 min) and under ultraviolet irradiation (90 min) in the presence of samples with different TiO_2_ mass contents. **d** The dependence of MB decolouration percentage on TiO_2_ mass content (%) by AD and photocatalytic (PH) processes. Samples are identified by percentage of TiO_2_ followed by method name (e.g. 50M1).

Figure 5a shows the degradation profile of the MB dye under dark conditions. From these data, the dependence of the MB decolouration percentage on the TiO_2_ mass content (%) by AD was calculated (Fig. 5b). The adsorption capability of samples with low TiO_2_ loading synthesised by M1, in particular of sample 25M1, was higher than that of samples with higher TiO_2_ mass content. By contrast, in samples synthesised by M2, the reverse was true, probably because of the large surface area of TiO_2_ prepared by this method.

**Fig. 6:**
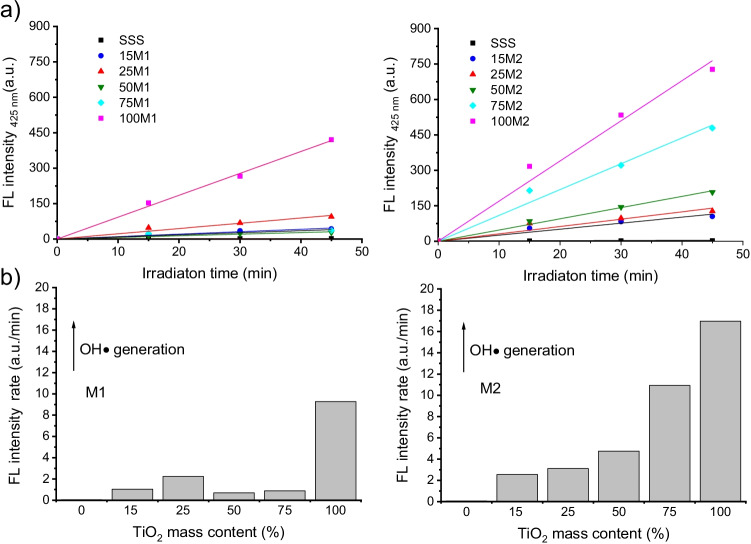
a Time dependence of the fluorescence (FL) intensity at 425 nm of raw stainless steel slag (SSS) and SSS/TiO_2_ composites fabricated by methods (left) M1 and (right) M2. Samples are identified by percentage of TiO_2_ followed by method name (e.g. 50M1). **b** Dependence of fluorescence intensity linear rate on TiO_2_ mass content (%).

Figure 5c shows the MB decomposition under UV irradiation after 30 min of adsorption in the dark. Because of the photoactivation of TiO_2_ nanoparticles, enhanced dye degradation was observed when the UV light was turned on, except in sample 25M1, the decolouration percentage of which remained almost constant. This sample does not show photocatalytic activity, despite evidence of UV light absorption and suitable band energies (Figs. 3 and 4): the anomalous result could be attributed to pore blockage by MB adsorbed molecules in the initial stage, limiting the light from arriving at the TiO_2_ active centres. Based on these data, the dependence of the MB decolouration percentage on the TiO_2_ mass content (%) by PH and synergetic AD + PH was calculated (Fig. 5d). The photocatalytic performance of samples with low TiO_2_ loading synthesised by M1, especially 15M1, is much higher than that of samples with higher TiO_2_ mass content. These results provide evidence of the synergic capacities of M1 composites. For M2, the MB discolouration under light irradiation, like the adsorption, increased with increasing TiO_2_ concentration, confirming the role of SSS as a simple support.

The TA-FL method allows indirect determination of the photocatalytic performance of the samples without adsorption and thus elucidation of 25M1s irregular behaviour. Figure 6a shows the increase in fluorescence intensity at 425 nm with UV illumination time. Every sample exhibited an increase in intensity compared to SSS. The linear dependence of the fluorescence intensity rate with respect to the TiO_2_ mass content (%) is shown in Fig. 6b. Sample M1 showed less OH• generation than sample M2, in agreement with the position of their electronic bands (Fig. 4b). The production of OH• on M1 samples must be through O_2_•- mediated production, because H_2_O or OH- cannot be oxidised. Further observation confirms the higher OH• generation of the 25M1 sample in comparison with the other composites synthesised by M1.

**Fig. 7:**
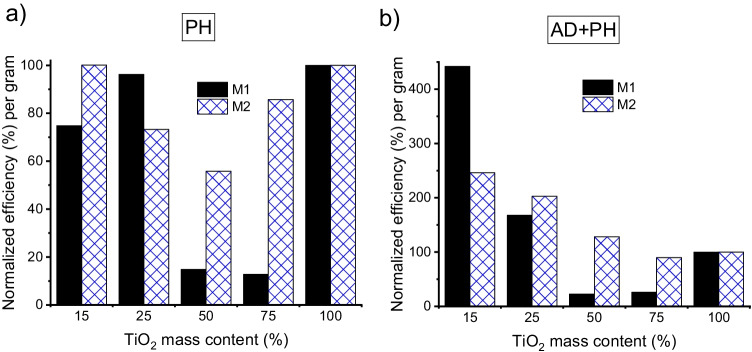
Photocatalytic efficiency of samples prepared by methods M1 and M2 in the case of **a** photocatalysis (calculated from terephthalic acid fluorescence data) and **b** combined adsorption and photocatalytic processes (calculated from measured degradation of methylene blue)

In order to compare the photocatalytic efficiency of each sample on the basis of the raw material used, the results were normalised as a function of the amount of TiO_2_ (grams), assigning 100% to bare TiO_2_ in both methods (100M1 and 100M2). As shown in Fig. 7a, sample 25M1 achieved almost the same photocatalytic efficiency per gram as pure TiO_2_, with sample 15M1 having a high efficiency of approximately 75%; 50M1 and 75M1 did not exhibit suitable performance. In the case of M2, 15M2 reached the same efficiency per gram as bare TiO_2_; the sample with 50% SSS exhibited the worst behaviour. The efficiency improvement with lower TiO_2_ content in the composite samples can be related to the better dispersion of TiO_2_ in the matrix. Particle aggregation and agglomeration influence the optical properties of materials; therefore, their ability to absorb and scatter incoming radiation also affects their photocatalytic activity (Pellegrino et al. 2017). Moreover, the introduction of SSS into composite samples results in a shift towards negative potentials relative to that of bare TiO_2_; this improves the oxidation photocatalytic efficiency.

The AD-PH combination procedure (Fig. 7b) led to even more favourable results in samples with lower TiO_2_ content: 15M1 and 25M1 were more than 4.4 and 1.6 times as efficient, respectively, as bare TiO_2_. Adsorbent photocatalyst (SSS-TiO_2_) may lead to synergetic effects by the formation of a common contact interface between the different solid phases, in which SSS acts as an efficient adsorption trap for organic compounds. The pollutant is then more efficiently transferred to the TiO_2_ surface, which is more rapidly photodegraded.

### Discussion of adsorption and photocatalytic mechanism

The MB decolourisation mechanism of SSS/TiO_2_ composites involves the adsorption of SSS and the photocatalytic degradation of TiO_2_. The MB molecule (~ 1.38–1.44 nm long (Souza et al. 2006, Dotto et al. 2015) and 0.95 nm wide (Jia et al. 2018)) is smaller than the pores in SSS, which can therefore adsorb MB. Moreover, the pores are numerous and have a large surface area. The photocatalytic oxidation of dye molecules can proceed by reaction with reactive oxygen species, such as superoxide anion radical (O_2_•^–^), hydrogen peroxide (H_2_O_2_), singlet oxygen (^1^O_2_) and hydroxyl radical (OH•) or with photogenerated holes, which are involved in a rapid adsorption–desorption equilibrium at the TiO_2_–solution interface. Surface-trapped holes can be regarded as adsorbed OH•(Jimenez-Relinque and Castellote 2015). Upon UV–visible irradiation, TiO_2_ converts incoming photons into electron/hole pairs with energies above or equal to the bandgap (E_g_ ≈ 3.1, –3.2 eV, according to data obtained from DRS measurements). After electron–hole pair separation, only electrons or holes that migrate to the surface can successfully drive oxidation reactions. Once there, the photogenerated holes react directly with the MB molecules or with H_2_O and OH- to produce OH•, while the photogenerated electrons react with O_2_, producing O_2_•-. Secondary photo-redox steps generate other transient reactive oxygen species, notably OH•, the most powerful oxidising species and the one primarily responsible for photodegradation processes in aqueous solution (Jimenez-Relinque and Castellote 2015, Jimenez-Relinque and Castellote 2018).

The process of decolourisation by synergetic adsorption photocatalysis can be divided into the following steps. First, the MB molecules are adsorbed onto the SSS structure, where the pore structure increases the opportunity of contact between the TiO_2_ surface and MB, accelerating photodegradation. Second, photoactivated TiO_2_ drives oxidation reactions by reactive oxygen species or holes that oxidise MB molecules and their intermediates.

## Conclusions

In this study, economical and environmentally friendly SSS-TiO_2_ composites derived from metallurgical waste were successfully synthesised by simple precipitation–calcination routes. Samples with different TiO_2_ loadings were prepared using a titanium isopropoxide precursor (M1) or commercial TiO_2_ nanoparticles (M2). The absolute photocatalytic activity was the highest for the bare TiO_2_ composite samples, but the photocatalytic efficiency normalised as a function of the amount of TiO_2_ demonstrated that, for both methods, composite samples with 15–25% TiO_2_ content achieved the same or almost the same efficiency per gram as pure TiO_2_. The adsorption–photocatalysis combination led to even more favourable results on samples with lower TiO_2_ content, 15M1 and 25M1 exhibiting higher efficiency than bare TiO_2_. This enhanced capability is due to the integration of TiO_2_ into the crystalline structure of SSS. In the mixed system, the adsorbent photocatalyst (SSS-TiO_2_) may lead to a synergetic effect by the formation of a common contact interface between the different solid phases, with SSS acting as an efficient adsorption trap for organic compounds. The pollutant is then more efficiently transferred to the TiO_2_ surface and rapidly photodegraded. Moreover, this optimal TiO_2_ loading may improve the UV absorption ability and enhance the photocatalytic oxidation capability. Our work demonstrates the possibility of simultaneous adsorption and photocatalysis for dye wastewater treatment of TiO_2_ combined with steel slag waste materials. Thus, it could offer novel solutions to the management of both metallurgical waste and water pollution.

## Supplementary Information

Below is the link to the electronic supplementary material.

Supplementary file1 (DOCX 16 KB)

## Data Availability

Not applicable.
