# Supplementary material for: Synergetic adsorption–photocatalysis process for water treatment using TiO2 supported on waste stainless steel slag
Source: Environ Sci Pollut Res Int. 2022 Feb 2;29(26):39712–22. doi: 10.1007/s11356-022-18728-8 (PMC9120099; doi:10.1007/s11356-022-18728-8)
Supplement: Supplementary file 1 — Supplementary file1 (DOCX 16 KB) [file 11356_2022_18728_MOESM1_ESM.docx]

**Electronic Supplementary Information**

**Synergetic Adsorption-Photocatalysis Process for Water Treatment Using TiO_2_ supported on Waste Stainless-Steel Slag**

Eva Jimenez-Relinque*, Siaw Foon Lee, Lorenzo Plaza, and Marta Castellote

Eduardo Torroja Institute of Construction Sciences, IETcc, CSIC, Serrano Galvache 4, 28033 Madrid, Spain

*Corresponding author: Eva Jiménez Relinque

E-mail address: eva.jimenez@csic.es

Table S1. pH, conductivity and leached contents from stainless steel slags (SSS) and chemical limits defined in the Italian, French and German regulations.

| Parameters | SSS sample * | | Italian^a^ | French^b^ | German^c^ |
| --- | --- | --- | --- | --- | --- |
| pH | | 11.38 | 5-12 | - | - |
| Conductivity(μS/cm) | | 1500 | - | - | - |
| Chemical compounds (mg/l) | | | | | |
| Cd | <0.05 | | 5 | 0.2 | 0.1-0.5 |
| Cu | <0.010 | | 0.05 | 0.5 | 5-10 |
| Ni | <0.020 | | - | 0.5 | 1-2 |
| Cr | 0.033 | | 0.05 | 0.5 | - |
| Mn | 0.09 | | - | - | - |
| Pb | <0.020 | | - | 0.5 | 1-2 |
| Zn | <0.05 | | 3 | 2 | 5-10 |
| Fluorides | <1 | | 1.5 | - | - |
| Chlorides | 10 | | 100 | - | - |
| Sulphates | 39.1 | | 250 | - | - |
| DOG | 26.2 | | 30 | - | - |

*medium value in 2 fractions; ^a^ limit value for steel slag waste recovery, ^b^ Maximum values for non-ecotoxic residues; ^c^ Interval limits/minimum values for hazardous residues; DOG: Dissolved organic carbon.
